# Sustained complete remission with third-line nivolumab in advanced renal cell carcinoma: a case report

**DOI:** 10.1186/s13256-026-05886-3

**Published:** 2026-02-25

**Authors:** Zsófia Küronya, Enikő Lénárt, Edina Soós, Lajos Géczi, Krisztina Biró

**Affiliations:** Department of Genitourinary Medical Oncology and Clinical Pharmacology, National Institute of Oncology, Ráth György Str. 7-9, 1122 Budapest, Hungary

**Keywords:** Renal cell carcinoma, Immunotherapy, Nivolumab, Complete response, Immune-related adverse events, Hungary

## Abstract

**Background:**

Renal cell carcinoma is a challenging malignancy that often necessitates advanced systemic therapies. Immunotherapy with checkpoint inhibitors has emerged as a cornerstone for managing advanced renal cell carcinoma. This case highlights the clinical course, treatment response, and management of complications in a patient with advanced renal cell carcinoma treated with immunotherapy.

**Case presentation:**

A 51-year-old white male patient presented with dizziness, leading to a diagnosis of advanced renal cell carcinoma involving a large left renal mass, pulmonary metastases, and retroperitoneal lymphadenopathy. After radical nephrectomy in 2016, histopathology confirmed T3a clear cell carcinoma with vascular invasion. Initial systemic treatment included targeted therapy with sunitinib, followed by axitinib owing to disease progression. Nivolumab, a programmed death-1 inhibitor, became available in Hungary in 2018 and was initiated as third-line therapy in mid-2018. The patient has remained disease-free without any therapy for the past 27 months, with follow-up imaging through September 2025 confirming sustained complete remission first documented in October 2020, amounting to nearly 5 years of continuous complete remission. The patient experienced manageable immune-related adverse events, including nephritis and colitis, which were treated with corticosteroid therapy. Routine imaging and clinical assessments confirmed no evidence of recurrence as of September 2025. This case underscores the efficacy and durability of immune checkpoint inhibitors in advanced renal cell carcinoma.

**Conclusion:**

This case exemplifies the long-term benefit of immunotherapy in advanced renal cell carcinoma, emphasizing the importance of vigilant monitoring for immune-related adverse events, sustained follow-up, and the potential for extended therapy-free remission.

## Background

Renal cell carcinoma (RCC) accounts for approximately 2–3% of adult malignancies. While localized disease can often be managed with surgical resection, metastatic RCC (mRCC) has a poor prognosis, with 5-year survival rates under 10% in untreated patients. The advent of immune checkpoint inhibitors (ICIs), such as nivolumab, has significantly transformed the therapeutic landscape of RCC by offering durable responses in a subset of patients. These agents have emerged as cornerstones in advanced RCC management, as outlined in a comprehensive review by Xu *et al*. [1]. This case highlights the use of nivolumab in a resource-limited setting, where the availability of advanced therapies is often delayed. The patient’s sustained complete remission further underscores the potential efficacy of ICIs. Current international guidelines recommend immune checkpoint inhibitor combinations (immuno-oncology–immuno-oncology [IO–IO]) or immunotherapy combined with tyrosine kinase inhibitors (immuno-oncology–tyrosine kinase inhibitors [TKIs]) as standard first-line treatment for metastatic renal cell carcinoma. In this modern therapeutic landscape, achieving a durable complete remission with third-line nivolumab has become increasingly uncommon, underscoring the relevance of this case. It is important to note that when this patient began systemic therapy in 2016, combination immunotherapy and IO–TKI regimens were not yet established or widely available, and sequential monotherapies represented standard practice at that time.

## Case presentation

A 51-year-old white male patient with a history of controlled hypertension, no history of smoking, and no family history of malignancy presented with persistent dizziness in February 2016.

Physical examination revealed a palpable mass in the left upper quadrant of the abdomen. Initial imaging revealed a large 10 cm × 9 cm × 10 cm lobulated mass in the left kidney with features consistent with renal cell carcinoma. Staging investigations revealed retroperitoneal lymphadenopathy and pulmonary nodules suggestive of metastases. The patient was classified into the International Metastatic RCC Database Consortium (IMDC) good prognostic group at presentation.

**Fig. 1:**
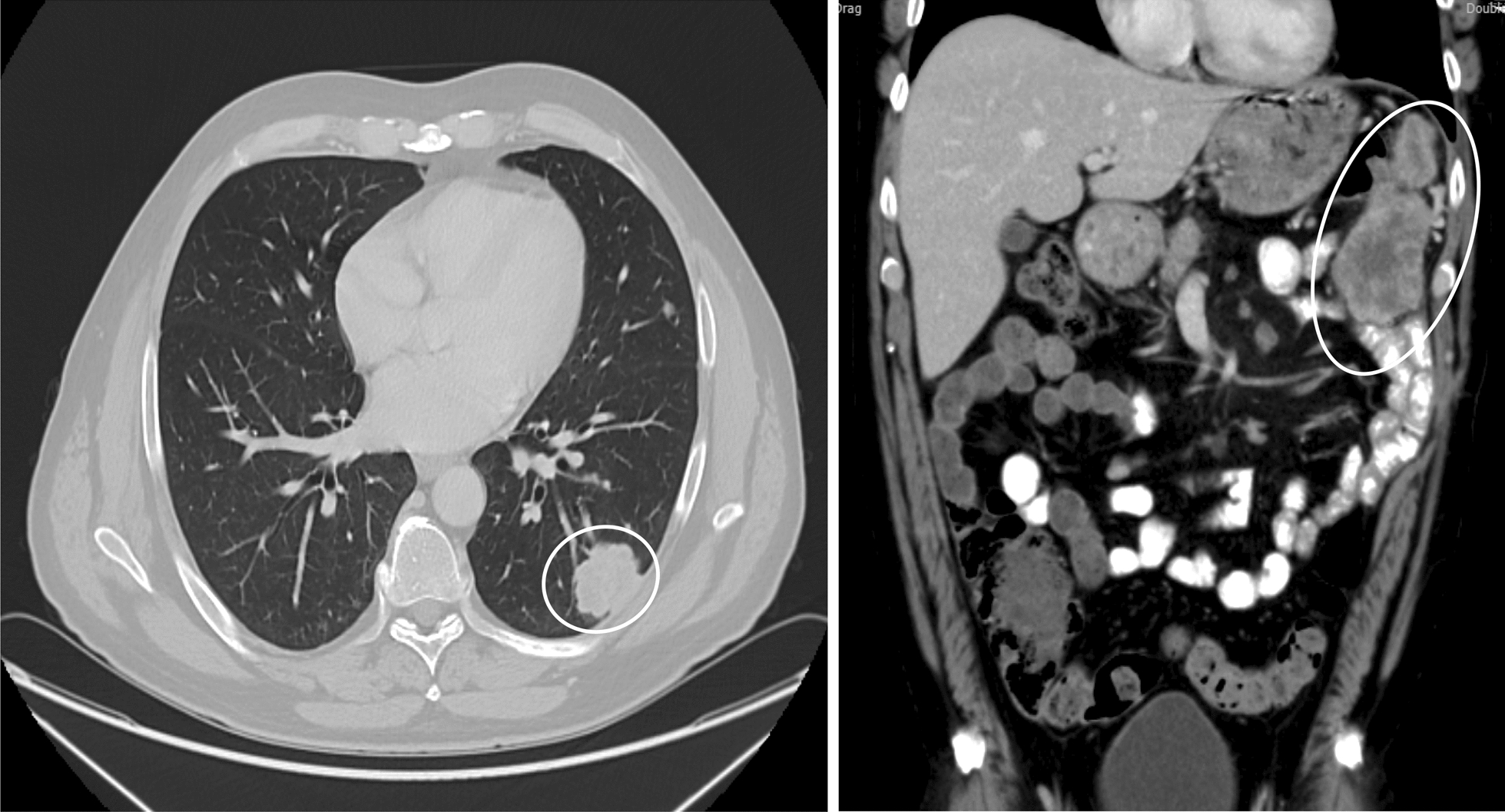
Baseline contrast-enhanced computed tomography images obtained in 2018 prior to nivolumab initiation. **A** Axial chest computed tomography demonstrating a subpleural intrapulmonary metastatic lesion with a lobulated contour in the left lung (highlighted by a circle). **B** Axial abdominal computed tomography showing a 3.0 cm × 2.4 cm metastatic mass in the lower segment of the spleen, with additional metastatic lesions detected around the spleen, between the intestinal loops, and beneath the abdominal wall (highlighted by ovals)

A radical nephrectomy was performed in March 2016, and histopathology confirmed T3a clear cell carcinoma with vascular invasion. Initial systemic therapy with sunitinib (starting July 2016) achieved partial response, but disease progression necessitated sequential treatments with axitinib (initiated in June 2017). However, progression continued, with multiple pulmonary and abdominal metastases identified by early 2018, with pulmonary metastases shown in Fig. 1A and abdominal metastases in Fig. 1B.

Nivolumab became available in Hungary in mid-2018, and treatment was initiated shortly thereafter, in August 2018. The patient initially received biweekly doses of 240 mg nivolumab. However, in 2019, the dosing schedule was adjusted to 480 mg every 4 weeks, in line with standard treatment protocols. Imaging by October 2018 demonstrated significant regression of pulmonary and abdominal lesions, with no new metastases. At the initiation of nivolumab therapy, physical examination revealed a palpable resistance of approximately 3 cm × 4 cm at the site of the nephrectomy scar. This palpable tumor gradually diminished in size during treatment and eventually disappeared.

At the initiation of nivolumab therapy, physical examination revealed a palpable resistance of approximately 3 cm × 4 cm at the site of the nephrectomy scar.

Computed tomography (CT) imaging confirmed that this palpable resistance corresponded to metastatic disease, with abnormal soft-tissue lesions in the left renal fossa, spleen, pancreatic tail region, and along the peritoneum, as well as disseminated pulmonary nodules. This palpable tumor gradually diminished in size during treatment and eventually disappeared.

**Fig. 2:**
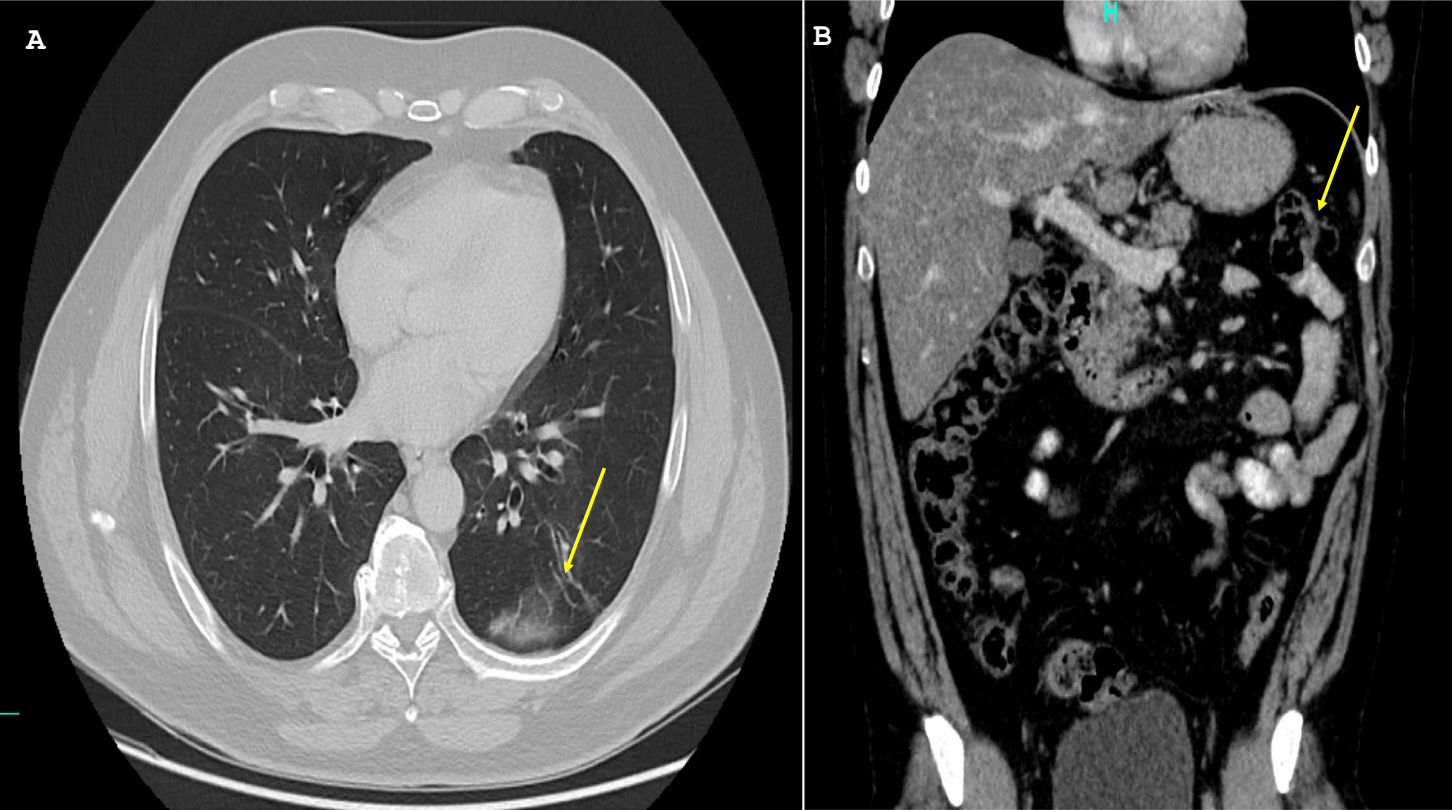
Follow-up contrast-enhanced computed tomography images obtained in 2020 demonstrating a complete radiologic response. **A** Axial chest computed tomography showing complete radiologic response of the previously identified pulmonary metastasis (arrow). **B** Axial abdominal computed tomography demonstrating complete radiologic response of the previously described abdominal metastatic lesions (arrow)

Continued follow-up through late 2020 confirmed complete response, with resolution of all pulmonary lesions, as shown in Fig. 2A, and complete abdominal remission in Fig. 2B.

Nivolumab therapy was initiated in August 2018 and discontinued in August 2023. A radiologically confirmed complete remission was first documented in October 2020, and the patient maintained this remission for nearly 3 years. Given the absence of any evidence of active disease on repeated imaging studies and on the basis of emerging expert recommendations that prolonged immunotherapy may be safely discontinued in patients with durable complete remission, treatment was stopped following clinical evaluation. Since discontinuation, the patient has remained disease-free for 27 months, with follow-up imaging in September 2025 confirming ongoing complete remission, with an Eastern Cooperative Oncology Group (ECOG) performance status of 0.

**Fig. 3:**
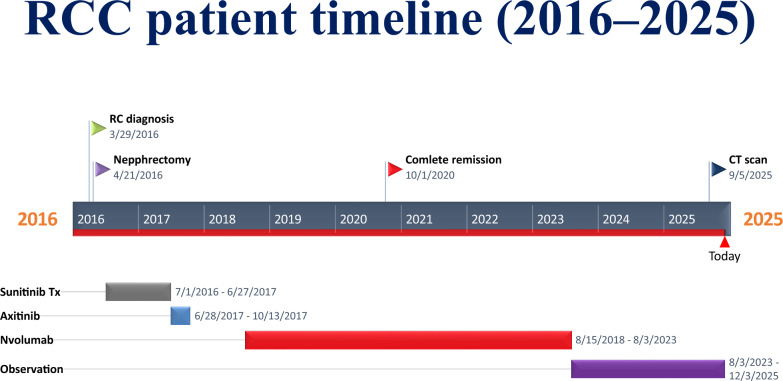
Flow chart showing the main events of the patient’s treatment

Fig. 3 shows the main events of the patient’s treatment.

The patient experienced immune-related adverse events (irAEs), including grade 2 nephritis and colitis, during the early phase of nivolumab treatment. The episode of nephritis was supported by elevated serum creatinine and mild proteinuria on urinalysis, while the colitis was identified clinically and corroborated by elevated inflammatory markers and abdominal imaging findings. The immunotherapy was temporarily withheld until the colitis and nephritis had completely resolved. The patient received systemic corticosteroids at a dose of 1 mg/kg/day of prednisolone, which was tapered over the course of approximately 1 month. No long-term sequelae were observed, and the patient tolerated the treatment well overall.

The patient reported that although the early immune-related adverse events were challenging, they were manageable with corticosteroid treatment. He expressed gratitude for the opportunity to receive immunotherapy and described the prolonged complete remission as life-changing, allowing him to resume normal daily activities without limitation.

## Discussion

This case highlights the transformative potential of immune checkpoint inhibitors (ICIs) in metastatic renal cell carcinoma (mRCC), especially when access to immunotherapy may be delayed. Nivolumab, a programmed death-1 (PD-1) inhibitor, demonstrated durable responses and improved overall survival in the pivotal CheckMate 025 trial [2]. These results provide an important context for understanding the remarkable response observed in our patient.

To contextualize our case within the current evidence base, findings from the CheckMate 025 trial are particularly relevant. In this study, 72% of patients received nivolumab as second-line therapy and 28% as third-line therapy, demonstrating broad applicability across different treatment lines in advanced RCC. The overall response rate (ORR) with nivolumab was 23%, including a 1% complete response (CR) rate and 22% partial response (PR) rate. Notably, nearly half of responding patients maintained durable responses, with 31% having ongoing responses lasting 12 months or longer [2]. These findings support the potential for meaningful clinical benefit even in later-line settings.

Despite these encouraging results, one of the major challenges in RCC treatment is the lack of reliable biomarkers to predict response to immunotherapy. Unlike certain malignancies where biomarker-based decisions are standard, RCC exhibits significant biological heterogeneity and lacks established predictive markers. As a result, treatment sequencing often relies on clinical judgment rather than molecular guidance, as illustrated by the use of nivolumab as a third-line therapy in this case.

In recent years, several studies have suggested that immune-related adverse events (irAEs), such as colitis or hepatitis, may be associated with improved treatment responses to ICIs [3, 4]. The development of grade 2 nephritis and colitis in our patient is consistent with these observations and further supports the association between irAEs and durable treatment response. Prompt recognition and corticosteroid management allowed the continuation of therapy without major interruption, aligning with American Society of Clinical Oncology (ASCO) guidelines recommending early intervention to maintain treatment continuity [5].

The patient’s extended remission without therapy is particularly noteworthy, suggesting durable immune-mediated tumor control. This aligns with recent pooled analyses, including those by Chang *et al*., demonstrating significant treatment-free survival benefits with IO + TKI combinations [6] and with studies reporting similar findings for the ipilimumab + nivolumab regimen [7]. Although a few similar cases have been reported in literature—including reports of complete remission achieved with third-line nivolumab—such durable responses remain exceedingly rare. For example, Kurahashi *et al*. (2020) documented a patient achieving complete remission after third-line nivolumab [8], and another case described successful nivolumab therapy in a patient with RCC and bone metastases [9]. Taken together, these findings underscore both the therapeutic potential of ICIs and the exceptional nature of this patient’s prolonged, treatment-free complete remission.

## Conclusion

This case highlights the efficacy of nivolumab in achieving sustained CR in advanced RCC, even in the context of limited access to novel therapies. The patient’s exceptional response, including over 27 months of therapy-free remission, underscores the potential for ICIs to induce long-term disease control. These findings support the integration of checkpoint inhibitors into standard mRCC management and reinforce the importance of personalized follow-up strategies.

## Data Availability

The datasets used and/or analyzed during the current study are available from the corresponding author upon reasonable request.

## Abbreviations

RCC: Renal cell carcinoma
mRCC: Metastatic renal cell carcinoma
PD-1: Programmed death-1
irAE: Immune-related adverse event
IMDC: International Metastatic RCC Database Consortium
ECOG: Eastern Cooperative Oncology Group
CR: Complete response
PR: Partial response
ORR: Overall response rate
IO: Immuno-oncology
TKI: Tyrosine kinase inhibitor
ICI: Immune checkpoint inhibitor
ASCO: American Society of Clinical Oncology
